# Salmonid Antibacterial Immunity: An Aquaculture Perspective

**DOI:** 10.3390/biology9100331

**Published:** 2020-10-11

**Authors:** Shawna L. Semple, Brian Dixon

**Affiliations:** Department of Biology, University of Waterloo, Waterloo, ON N2L 3G1, Canada; slsemple@uwaterloo.ca

**Keywords:** aquaculture, salmonids, bacterial pathogens, comparative immunology, adjuvants, vaccines, immunological memory, bacterial pathogenesis, teleost

## Abstract

**Simple Summary:**

Capture fisheries are reaching their limit, so the increasing demand for fish protein can only be met through aquaculture. One attractive sector within this industry is the culture of salmonids, which are a) uniquely under pressure due to overfishing and b) the most valuable finfish per unit of weight. The culture of these animals is threatened by many diseases, some caused by bacteria, which can result in large financial losses for fish farmers. Unfortunately, the current methods for the control of aquatic bacterial diseases are either unsustainable (antibiotics) or not very effective (vaccines). This is primarily due to a lack of knowledge surrounding the successful immune function of fish. To improve vaccine design and other methods of control, a deeper understanding of fish immunology is essential. This review highlights the current understanding of fish antibacterial immunity in the context of salmonid culture. Additionally, the successes and shortcomings of current methods used to combat bacterial diseases in salmonid aquaculture will be addressed. Improving our understanding of the salmonid immune system will help to reduce aquaculture losses in the future.

**Abstract:**

The aquaculture industry is continuously threatened by infectious diseases, including those of bacterial origin. Regardless of the disease burden, aquaculture is already the main method for producing fish protein, having displaced capture fisheries. One attractive sector within this industry is the culture of salmonids, which are (a) uniquely under pressure due to overfishing and (b) the most valuable finfish per unit of weight. There are still knowledge gaps in the understanding of fish immunity, leading to vaccines that are not as effective as in terrestrial species, thus a common method to combat bacterial disease outbreaks is the use of antibiotics. Though effective, this method increases both the prevalence and risk of generating antibiotic-resistant bacteria. To facilitate vaccine design and/or alternative treatment efforts, a deeper understanding of the teleost immune system is essential. This review highlights the current state of teleost antibacterial immunity in the context of salmonid aquaculture. Additionally, the success of current techniques/methods used to combat bacterial diseases in salmonid aquaculture will be addressed. Filling the immunology knowledge gaps highlighted here will assist in reducing aquaculture losses in the future.

## 1. The Impact of Global Aquaculture

Given that both fresh- and saltwater account for 72% of Earth’s surface area, it was only a matter of time before aquatic environments became the new frontier for agriculture. Because the majority of food animals are currently raised on land, it is unsurprising that insights or advancements in aquatic animal husbandry have lagged behind those of terrestrial species. As the global population increases, and given the limited availability of productive land, the necessity of utilizing aquatic habitats for animal food production is clear. Additionally, due to their high polyunsaturated fatty acid content [1], many aquatic species provide an alternative and heart healthy protein source in an age when cardiovascular disease is the leading cause of death worldwide [2]. For these reasons and more, global interest for fish protein is high. It is so high, in fact, that fisheries cannot meet the global demand while also adhering to the harvesting restrictions that are currently in place [3]. This places a burden on wild populations because effective enforcement of these restrictions is logistically difficult.

The culture of aquatic species, or aquaculture, can provide an alternative to alleviate some of the pressure on wild populations. For many aquatic species, this culture production is in its nascent form, meaning that time will be required to understand and optimize these industrial practises. One manifestation of the issues faced by aquaculture is the increased prevalence of infectious disease, including bacterial pathogens. Bacteria are able to take advantage of novel, high density farm environments and thrive. This results in many of these prevalent microorganisms becoming opportunistic pathogens in aquaculture settings. Obtaining a deeper understanding of bacterial diseases that impact aquaculture, as well as what constitutes an effective immune response in relevant hosts, is invaluable for the improvement of this industry. For the purposes of this review, the term “aquaculture” will be refer to the culture of finfish species.

The utilization of aquatic environments means that some of the difficulties confronted by fish farmers are very different when compared to their terrestrial counterparts. Common sources of financial losses include environmental/husbandry (algal blooms, temperature oscillations, hypoxia, supersaturation, etc.), chemical (nitrogen fluctuations, pH variation, etc.), predation, escapees and infectious disease [4]. Many of these problems can result in devastating financial losses, but few compare to the consistent annual losses derived from infectious disease. In 2014, of the $70 billion dollars of aquaculture product that was destined for human consumption, 10% of this was lost due to infectious disease [5,6]. Though there are many different types of infectious agents that contribute to the significant financial losses experienced in aquaculture, this review will focus on bacterial diseases of salmonid culture, the most valuable finfish species per unit of weight within this global industry [7].

## 2. Common Bacterial Diseases in Salmonid Culture

Cultured salmonids are susceptible to many bacterial pathogens (Table 1). The stress induced by conditions such as overcrowding, temperature fluctuations and excessive handling can result in normally benign microorganisms becoming opportunistic pathogens [8]. The aquatic environments in which these animals reside are known to support the growth of bacteria for long periods of time. Though not immediately causing infection, these opportunistic bacterial pathogens can survive independently of their hosts [9,10,11]. When animals are stressed, these microorganisms are well situated to become major impediments for aquaculture. The ectothermic nature of fish means that these animals have no control over their body temperature as it is simply a representation of their surrounding environment [12]. As a result, there are different opportunistic bacterial pathogens that have taken advantage of the variety of temperature niches. For fish farmers, this has made management and prevention strategies difficult as there is a large degree of variability in the route of entry, virulence factors, disease presentations and pathologic cycle between the various bacterial pathogens (reviewed in [13,14]). The significance of bacterial pathogens in salmonid aquaculture, combined with the extensive knowledge gaps regarding host immunity, means that further investigation of the salmonid immune system could prove invaluable.

## 3. Teleostean Immunity: Our Current Understanding of the Antibacterial Response

Despite their divergence occurring 320–350 million years ago (reviewed in [15]), the teleost immune system contains many components comparable to what is known in mammals. Bony fishes are divided into the Sarcopterygii (the lobe-finned fish) and the Actinopterygii (ray-finned fish) to which Teleostei (Greek for “complete bone”) belongs (reviewed in [16]). Salmonids, which will be heavily emphasized in this review, are members of Teleostei. The teleosts comprise 95% of surviving fish species, which represents approximately half of all extant vertebrate species [17]. The remarkable success of this class, along with their ability to thrive in a wide range of environments, suggests that teleosts developed an impressive immune arsenal to counter pathogen challenge. Much like the highly studied mammalian model, the immune system of teleosts can be separated into two main branches: the innate and adaptive immune systems. The following information represents a brief summary of teleostean immune defenses that are common responses to bacterial pathogens. For a more in-depth overview of known teleostean immune mechanisms, please see [18,19,20,21,22,23]. 

### 3.1. Innate Immunity of Fish

Due to the limitations of teleost adaptive immunity (i.e., slow initiation, limited antibody repertoire, etc.), the burden of preventing and combatting infectious agents falls heavily to the innate immune system. Fish have been shown to have all of the mammalian aspects generally associated with innate immunity including physical barriers (skin and mucous membranes), humoral parameters (complement, natural antibody, toll-like receptors, etc.) and cellular components (phagocytosis, NK cells, etc.). As the first line of defense, it is not surprising that the majority of the broad-spectrum parameters of innate immunity are highly conserved across species and taxa. In all jawed vertebrates, the innate immune system features a rapid defensive response towards invading pathogens and tissue damage. However, it cannot provide well-directed, specific protection from individual pathogens or long-term immunological memory.

#### 3.1.1. Cells of Innate Immunity

All of the innate immune cells that are observed in mammalian blood are also present in the blood of teleosts (monocytes, neutrophils, basophils and eosinophils), albeit at very different circulating concentrations. Neutrophils, basophils and eosinophils are referred to together as granulocytes and are aptly named due to the presence of cytoplasmic granules. These are filled with enzymes and host defense peptides that can support immune responses during infections and/or allergic reactions (reviewed in [24,25,26,27]). Monocytes patrol the blood, contributing to inflammation, immune defenses and homeostasis by clearing pathogens and cellular debris. Additionally, monocytes can enter tissues and differentiate into macrophages or dendritic cells (DCs) to replenish these important immune cells (reviewed in [28]). Of these peripheral blood leukocytes, only monocytes, eosinophils and neutrophils are phagocytic. When it comes to cell numbers, the granulocytes are the most prevalent circulating WBC in mammals and represent 45–65% of this population, 92% of which are neutrophils. Meanwhile, monocytes make up only 8% of total mammalian WBCs (reviewed in [29,30]). In comparison, granulocytes comprise just 2–3% of teleostean WBCs, while monocytes are merely 0.1% [31,32]. Despite differences in circulating concentrations, the function of these WBCs appears to be conserved between the two taxa. 

As the principal phagocytic cells in fish, macrophages are considered one of the most important contributors to the innate immune defenses of these animals. Though macrophages can derive from monocytes (reviewed in [28]), this happens relatively infrequently [33]. Instead, recent evidence in mammals has shown that these cells are present in embryonic tissues (yolk sac and fetal liver) prior to hematopoiesis and can then persist as self-maintaining populations to perform organ specific functions (reviewed in [34]). Although further investigation is required, recent work with zebrafish has shown that tissue macrophages are present throughout adulthood even when adult hematopoiesis is absent [35]. This indicates that fish may also have tissue resident, self-maintaining macrophages. Functionally, macrophages are armed with many pattern recognition receptors (PRRs) enabling these cells to detect a multitude of pathogen associated molecular patterns (PAMPs) wherein strong binding will initiate phagocytosis of the foreign entity (reviewed in [36]). Once ingested, macrophages can rapidly kill foreign invaders through the production of toxic reactive intermediates and phagolysosomal acidification (reviewed in [19]). Besides their antimicrobial function, these cells are also able to present antigens to T cells and, depending on the surrounding stimuli, can orchestrate the appropriate immune response via cytokine secretion (reviewed in [19]). Finally, once an immune reaction has ceased, the phagocytic function of macrophages is critical for maintaining tissue homeostasis by clearing cellular debris (reviewed in [37]). The dynamic and heterogeneous nature of macrophages means that these cells can be distinguished depending on the source of activation and the resultant differences in cellular function, referred to as polarization (reviewed in [38,39]). In fish, the M1 macrophage polarization state is characterized the best and appears to serve a vital role in host protection against bacterial pathogens (reviewed in [18,19]). As a result, vaccination efforts for aquatic bacterial pathogens should focus on tailoring the teleostean immune response to stimulate the polarization of the M1 macrophage phenotype. 

#### 3.1.2. Pattern Recognition Receptors (PRRs)

A hallmark of innate immunity is the recognition of conserved, nonspecific molecules that are associated with infectious agents and/or cellular damage. These molecules make patterns that the host can recognize. This is accomplished by germline-encoded receptors known as pattern recognition receptors (PRRs) that are found on/within many different cell types. These receptors are collectively capable of binding to many different PAMPs, ensuring that the immune system will be notified when host barriers are breached. Upon binding to their associated ligands, a signalling cascade is initiated to stimulate transcription factors (such as NF-κΒ, AP-1, and NFAT), leading to the upregulation of genes involved in inflammatory responses so that the origin of the PAMP can be effectively opposed (reviewed in [40,41,42]). To date, there are six classes of PRRs which are categorized based on shared structural elements: toll-like receptors (TLRs), C-type lectin receptors (CLRs), NOD-like receptors (NLRs), AIM2-like receptors (ALRs), RIG-I-like receptors (RLRs) and cGAS/STING receptors (reviewed in [41]). Though not as well understood as their mammalian counterparts, receptors from all but the ALRs have been identified in teleosts [43,44,45,46,47]. Although there are some similarities between mammalian and teleostean PRRs, some discrepancies do exist. The substantial differences in habitat, life cycle, genome structure and reproductive strategies between these two taxa may provide some explanations. Perhaps further study of teleostean PRRs would yield insight regarding which specific facets can influence the evolutionary conservation of these molecules.

Of the different PRR classes, TLRs were the first discovered and are also considered to be the best characterized in both mammalian and teleostean models. At least 20 different TLRs have been found in more than a dozen fish species [48,49], but direct evidence of ligand specificity has only been shown in TLR2, TLR3, TLR5M, TLR5S, TLR9, TLR21, and TLR22 (reviewed in [48]). Five of these specifically bind and recognize PAMPs derived from bacteria such as peptidoglycan (TLR2, [50,51,52]), lipoteichoic acid (TLR2, [50,52]), flagellin (TLR5M and TLR5S, [53,54,55]) and CpG DNA (TLR9 and TLR21, [56,57,58,59]). Additionally, there are convincing results suggesting that some of the nonmammalian TLRs, such as TLR14, TLR18, and TLR25, may also be associated with sensing bacterial PAMPs [60,61,62,63]. Considering that these receptors are likely to target bacterial pathogens specific to the aquatic environment, they may represent effective targets for novel vaccine formulations in aquaculture. 

#### 3.1.3. Antimicrobial Peptides

Aside from notifying the immune system to the presence of a foreign entity, some aspects of innate immunity can act to directly destroy the infectious agent, as observed with antimicrobial peptides (AMPs). AMPs are a diverse class of highly conserved molecules that are produced as a first line of defense in all multicellular organisms, including teleosts. These small peptides (12–50 amino acids) are essential components of innate immunity capable of antimicrobial activity against a broad range of microbial pathogens (reviewed in [64]). Importantly, this also includes multi-drug resistant isolates [65,66] which have become a major concern in aquaculture. AMPs are often produced constitutively, but they can also be induced upon exposure to pathogens and/or other trauma [67,68]. Most AMPs are cationic amphipathic peptides that function by attacking the negatively charged membranes of microorganisms (reviewed in [69]). AMPs are characterized based on their secondary structures as one of four types: β-sheet, α-helix, extended or loop. Of these four types, β-sheet and α-helix are the most prevalent (reviewed in [70]). Functionally, they can be described as either membrane disruptive AMPs, inducing membrane permeabilization, or nonmembrane disruptive AMPs, which directly passage into cells and act on intracellular targets (reviewed in [71]). Besides the direct destruction of pathogens, AMPS can perform immunomodulatory functions in higher vertebrates (reviewed in [72]) and as a result are also called “host defense peptides” (HDPs). The potential immunomodulatory effects are diverse including stimulation of chemotaxis, immune cell differentiation, initiation of adaptive immunity and stimulation of both pro- and anti- inflammatory cytokines [73,74,75,76]. As many AMPs have multiple functions that can be both bactericidal and immunostimulatory in nature, there has been growing interest regarding their use in aquaculture as an alternative for antibiotics and/or as adjuvants (reviewed in [77,78,79]).

#### 3.1.4. Respiratory Burst Activity

**Figure 1:**
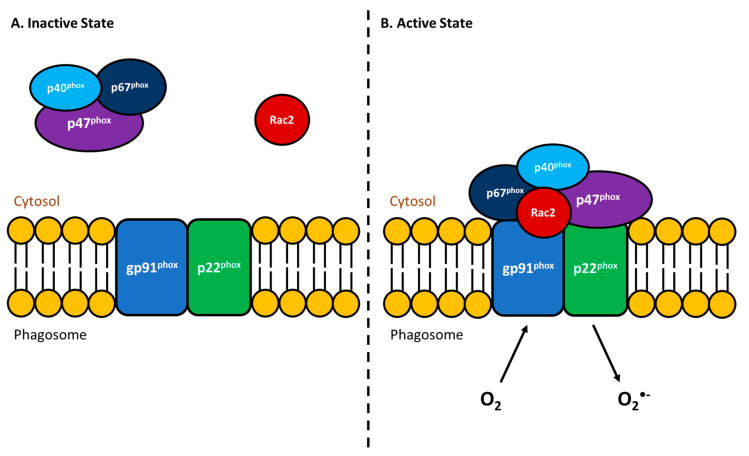
Schematic depiction of NADPH oxidase activation to enable the respiratory burst activity of phagocytes. The NADPH oxidase enzyme consists of six subunits. (**A**) When the phagocyte is in an inactive state, two of the subunits (gp91^phox^ and p22^phox^) are transmembrane components, while the remaining four components are cytosolic (p40^phox^, p47^phox^, p67^phox^ and Rac2). (**B**) Upon the phagocyte being activated by external stimuli, the four cytosolic components complex with gp91^phox^ and p22^phox^ to form the active NADPH oxidase. When in the active form, the now functional enzyme can convert molecular oxygen into superoxide anions to aid in the degradation or killing of that which was phagocytosed.

An essential immunological response for eliminating bacterial pathogens is the respiratory burst activity (RBA) of phagocytes. Following ingestion of foreign particles, these immune cells can kill most bacteria by producing reactive oxygen intermediates (ROI). The production of ROI requires NADPH oxidase (NOX), which catalyzes the conversion of molecular oxygen into superoxide anions (reviewed in [80,81]). Upon formation, the superoxide anion will then transform into further ROIs such as hydrogen peroxide, hydroxyl radical, and hypochlorous acid, all of which efficiently kill the phagocytosed microorganisms [82]. Inactive NOX consists of six subunits wherein gp91^phox^ and p22^phox^ are membrane proteins (Figure 1A) that together are known as flavocytochrome b_558_ (cyt b_558_, [83]). The remaining four regulatory subunits, (p40^phox^, p47^phox^, p67^phox^ and Rac2) normally exist in the cytosol (Figure 1A) but upon the activation of leukocytes by particulate stimuli, will translocate to the membrane and associate with cyt b_558_ (Figure 1B) to form the active oxidase (reviewed in [80,83]). It is well established that fish phagocytes possess all of these NADPH oxidase components as well as an RBA response comparable to that of mammals [84,85,86]. Additionally, this immune defense has been extensively studied in relation to bacterial pathogens of fish [87,88,89,90]. Given that the RBA in fish is not markedly influenced by temperature (reviewed in [91,92,93,94]), this innate immune response is an essential antibacterial mechanism for these poikilothermic organisms. 

### 3.2. Adaptive Immunity of Fish

All organisms have innate immune mechanisms, and while there are indications of adaptive immunity in invertebrates, this branch of the immune system appears to be an advancement that is specific to gnathostomes (jawed vertebrates). The adaptive immune system is remarkably flexible, capable of recognizing and initiating protective responses against specific foreign agents. Upon subsequent exposures, the adaptive immune system will remember antigens from a foreign invader, making it possible to mount a stronger and more efficient immune response (reviewed in [95,96]). Though delayed when compared to mammalian counterparts, the specificity of the teleostean adaptive immune system is essential for long-lasting immunological memory. As such, this branch of the immune system is critical for vaccine design, an enterprise in need of improvement for the aquaculture industry. 

#### 3.2.1. Cells of Adaptive Immunity

Much like in mammalian models, lymphocytes are considered the adaptive immune cells of fish. The two types of lymphocytes, T and B lymphocytes, represent the only cells capable of recognizing and responding specifically to an antigenic epitope. This antigen detection is based on compatibility with either the surface T cell receptor (TCR) or B cell receptor (BCR) depending on the lymphocyte. The genes that encode for these receptors undergo a series of DNA recombination events, providing them with an immense phenotypic diversity to improve the likelihood of antigen recognition (reviewed in [97]). Depending on the type of activation, T lymphocytes can produce cytokines to direct immune responses (CD4^+^ T cell, reviewed in [98]) or induce programmed cell death in virally infected cells (CD8^+^ T cell, reviewed in [99]). In comparison, B cells will transform into plasma cells following activation to produce antigen specific antibodies (reviewed in [100]). Though participating in a variety of different activities, the proficient function of both T and B lymphocytes is crucial for the success of the adaptive immune system. 

In mammals, lymphocytes represent the largest blood cell in diameter (8-10 µm) and account for approximately 20–40% of WBCs (reviewed in [29,30]). This is quite different from teleost species where lymphocytes are smaller in size (5–8 µm) and represent the dominant circulating leukocyte at 83–90% of total WBCs [31,32,101]. Regardless of the model system used, T and B lymphocytes appear identical when observed under a microscope, making it impossible to discern between the two without identifying cell surface markers. Though studied in detail for mammalian models, this was not possible in teleosts due to the absence of appropriate antibodies. Fortunately, with the recent development of antibodies specific to some of the cell surface markers on salmonid lymphocytes [102,103,104], comparative immunologists are able to finally start understanding adaptive immune functions in teleosts. 

#### 3.2.2. Major Histocompatibility (MH) Genes

**Figure 2:**
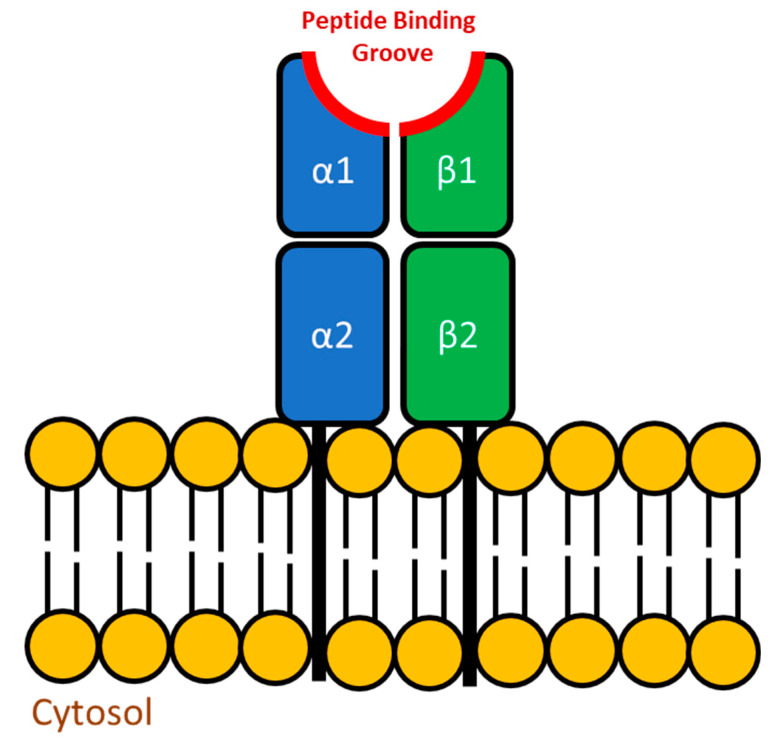
Structure of the MH class II molecule. The structure and function of the teleostean MH class II dimer is very similar to mammalian MHC class II. The molecule is a dimer, consisting of an alpha chain and beta chain, both of which have transmembrane regions. As outlined in red, both the α1 and β1 domains of this molecule have a hypervariable region that is part of the peptide binding groove. It is this region that is capable of binding to compatible antigens and presenting it to T cells.

To effectively combat the inevitable interaction with foreign entities, vertebrates have evolved two distinct antigen presentation pathways that, when paired with effective innate immune system activation, can stimulate long-term immunological memory. The major histocompatibility complex (MHC) molecules are critical in this important immune process yet the MHC gene equivalents in teleosts are not clustered on a single chromosome as they are in mammals so they are not considered to be a “complex”. Instead these genes can be found on more than one chromosome and as a result are simply referred to as Major Histocompatibility (MH) genes (reviewed in [105]). In the mammalian model, the endogenous antigen presentation pathway includes MHC class I molecules which are found within all nucleated cells. This pathway involves the processing of antigens from intracellular pathogens and their presentation via MHC class I to CD8^+^ cytotoxic T lymphocytes (reviewed in [106]). In comparison, the exogenous antigen presentation pathway uses MHC class II dimers which are generally only found on specific cell types, namely antigen presenting cells (APCs) that are capable of phagocytosis (reviewed in [107]). Once phagocytosed and processed, the antigens from extracellular sources are then loaded onto the MHC class II dimer and presented at the cell surface to CD4^+^ T lymphocytes (reviewed in [108]). In fish, the MHC equivalents function in an identical manner to what has been observed in mammals. Because this review focuses on bacterial pathogens, which are typically extracellular in nature, more emphasis will be placed on MH class II (Figure 2).

Per individual, the MH molecules play an important role by binding to and presenting well-matched peptides to appropriate T lymphocytes. The compatibility of these pathogen-derived antigens to MH molecules is controlled by allelic variation at the peptide binding groove. There are many possible alleles for the peptide binding region, and every individual within a species has a limited repertoire inherited from their parents in a Mendelian fashion (reviewed in [109,110]). The genetic polymorphism at the MH loci can provide more or less protection to pathogens, and thus these genes are believed to be under a strong selection pressure that is often governed by the surrounding habitat (reviewed in [111]). Regardless of the species, individuals that are heterozygous at MH loci are believed to be better protected because the resulting molecules should be able to bind to and present a more extensive collection of antigens [112,113]. In fish, this is supported by heterozygous individuals presenting less infection and/or mortality when challenged with an infectious agent [114,115]. Unlike what has been observed in some terrestrial species [116,117,118], specific MH alleles have not yet been shown to consistently predict resistance or susceptibility towards specific pathogens in fish, but perhaps more research is required.

#### 3.2.3. Antibody Development

Antibody development and production is of paramount importance in the humoral immune response of all jawed vertebrates, including bony fishes. This defense is particularly important when dealing with extracellular threats, as is the case with most bacterial pathogens. Antibodies prevent the growth and colonization of bacterial pathogens by neutralization, complement activation and/or opsonization to enhance phagocytosis (reviewed in [119]). To date, there are three known antibody classes in teleosts based on differences in their constant region: IgM, IgD, and IgT (reviewed in [120]). IgM was the first isotype discovered in teleosts and can be found on B cells as well as secreted in the serum or mucus as a tetramer (reviewed in [121]). The secreted form of teleost IgM is by far the most prevalent immunoglobulin in the serum and is responsible for systemic immunity in bony fishes (reviewed in [122]). At much lower concentrations, IgM is also present in the gut and skin mucosa. Similar to mammals, all mature IgM B cells in teleosts also express IgD, a class of antibody whose function is still not fully understood regardless of the model system used (reviewed in [120]). However, there have recently been slight improvements in elucidating mammalian IgD function (reviewed in [123]). Lastly, IgT is a recently discovered antibody isotype exclusive to bony fishes [124]. IgT is present within the serum as monomers, while forming tetramers in the gut mucosa. With concentrations of IgT in the gut mucosa being double those observed in the serum [102], it was believed that this Ig class likely had a vital role in mucosal immunity. This has since been heavily supported with numerous studies demonstrating the role of IgT in teleostean mucosal immunity [125,126,127]. When considering adaptive immune defenses that are important for combatting extracellular bacterial pathogens, effective antibody production and development is invaluable. 

Because teleosts do not have IgG as an antibody isotype (reviewed in [120]), any secondary antibody response observed is slight and uses a different approach than the canonical mammalian definition of immunological memory. This means that fish depend only on the low affinity but high avidity of IgM for repeated exposure to antigens, and thus a less intense secondary serum antibody response is often observed in these animals [128,129]. Additionally, fish do not appear to go through class switch recombination (CSR), despite having all of the necessary components and enzymes (i.e. AID, RAG1/2, Ikaros, TdT, etc.) required to complete this process (reviewed in [130]), due to the structure of their heavy chain genes. Interestingly, even though the catalytic domain of activation-induced (Cytidine) deaminase (AID) differs from tetrapods, when this enzyme is transfected into murine B cells it is still able to catalyze CSR [131]. Since fish do not appear capable of CSR, this finding revealed that the actual process of CSR must have evolved separately from the AID enzyme itself [131,132]. Protective responses have been observed in aquatic species for years following their initial exposure to antigens [133]. However, the requirements to consistently stimulate immunological memory/protection to selected pathogens must still be scientifically confirmed. 

Rather than developing memory B cells, it has been proposed that the most mature stage of B lymphocytes in teleosts are long-lived plasma cells (LLPCs). These cells reside within the head kidney, a primary immune organ of fish that is considered to be analogous to mammalian bone marrow. These LLPCs have been observed to secrete high amounts of antibody and appear to be the only detectable source of prolonged, high-titered antibodies [134]. However, the idea of ‘immunological memory’ is called into question because the antibody response in fish shows a poor affinity maturation and slow development of the secondary immune response, taking 3–4 weeks to initiate in fish [135]. This is a stark contrast to the rapid development observed within the mammalian paradigm of immunological memory, which likely influences the lack of efficacious vaccines for aquaculture production 

### 3.3. Cytokines

As described above, the immune system of vertebrates is a complex network connecting numerous cell types, barriers and specialized systems to prevent the entry and/or colonization of foreign entities within the host. The successful function of this multifaceted system depends on the ability of immune cells to migrate to and communicate with one another, a role fulfilled by extracellular mediators known as cytokines. Cytokines are a large family of small glycoproteins that are capable of acting in an autocrine, paracrine or endocrine fashion (reviewed in [136]). These soluble proteins play crucial roles in regulating inflammation, haematopoiesis, cellular movements and immune cell activation (reviewed in [137,138,139,140]). As such, cytokines act as an essential link between the innate and adaptive arms of the immune system. These small peptides ensure that vertebrates carry out an appropriate immune response to combat the specific threat. As a result, proper stimulation of cytokines could help increase the efficacy of vaccines and alternative treatment options for bacterial pathogens in aquaculture. 

#### 3.3.1. Chemokines

Chemotactic cytokines, or chemokines, are a large family of small proteins responsible for controlling the migratory patterns and positioning of immune cells (reviewed in [141]). Since the discovery of the very first teleostean chemokine in rainbow trout [142] several chemokines have been identified in other fish species, including Atlantic salmon, and zebrafish [143,144,145,146]. In both teleostean and mammalian models, interleukin (IL)-8, or CXCL8, is a highly studied chemokine due to its essential role in inflammation. Following its release by injured or infected tissue, IL-8 acts to recruit neutrophils to the site of injury. Upon arrival to these locations, neutrophils will then trigger proinflammatory responses, thereby attracting other important immune cells so that the threat can be eliminated [144,147]. IL-8 is an excellent indicator of immune activation as it shows not only the presence of these important immune cells, but also that they are actively engaged in clearing infections. IL-8 is an excellent indicator of immune system activation for research purposes. Additionally, effective manipulation of this cytokine could direct immune-based interventions, enabling a more accurate/efficient targeting of therapeutics.

#### 3.3.2. Proinflammatory Cytokines

Inflammation is an essential response in combatting tissue damage of any type, including harm associated with bacterial infection and/or associated toxins. Though there are many important components involved in an inflammatory response (reviewed in [148]), the initiation and perpetuation of inflammation is governed primarily by proinflammatory cytokines produced by damaged cells or responding immune cells. In mammalian models, the three classical proinflammatory cytokines are IL-1β, IL-6 and tumour necrosis factor (TNFα). Upon tissue damage, keratinocytes and fibroblasts release IL-1β to induce fever, T cell proliferation and increasing vascular permeability (reviewed in [149]). While this is occurring, resident mast cells (MCs) also degranulate in response to any mechanical trauma, releasing a wide variety of inflammatory mediators, including TNFα and IL-6 (reviewed in [150,151]). The released cache of mediators further aids in vascular permeability and the activation/recruitment of circulating immune cells which produce more proinflammatory cytokines. Leukocytes that are normally restricted to blood vessels will then gain access to the site of tissue injury and attempt to eliminate any invading targets (reviewed in [152]). If the inflammatory response is successful in clearing the threat, it is followed by a resolution and repair phase mediated mainly by tissue-resident and recruited macrophages that shift the response from a proinflammatory to anti-inflammatory one (reviewed in [153]). While fish immune systems are not as well characterized as mammalian equivalents, they possess all of the major proinflammatory cell types and cytokines (reviewed in [138,154]). 

#### 3.3.3. Anti-Inflammatory Cytokines

Though necessary for homeostatic maintenance, inflammatory responses can be quite damaging to surrounding tissues. This makes the control of such reactions essential for the day-to-day welfare of the host. Regulation and control of inflammatory responses requires a constant and ever-changing balance between proinflammatory and anti-inflammatory cytokines. Under normal circumstances, as the source of tissue damage is cleared, there are fewer stimuli available to induce a strong inflammatory response. This enables the constitutively produced anti-inflammatory cytokines to skew the reaction towards tissue repair mechanisms. Though there are several cytokines that are considered to have anti-inflammatory properties (reviewed in [136]), IL-10 is on that is believed to function solely as a potent anti-inflammatory cytokine. Produced by almost all leukocyte subsets, including T cells, B cells, macrophages and mast cells (reviewed in [155]), IL-10 plays a vital role in ensuring that both innate and adaptive immune responses cannot have a strong reaction unless a true threat is present. The importance of controlling inflammation through endogenous levels of IL-10 was made clear by Kühn et al. [156] when they generated IL-10 knockout mice. The IL-10 deficient mice spontaneously developed inflammatory enteritis, indicating that the mice were unable to prevent the inflammatory response towards their own commensal gut-associated bacteria [156]. Although the technology for producing knockouts in salmonid species is not yet available, the importance of IL-10 in the resolution of inflammation has been made clear in grass carp, rainbow trout and Atlantic salmon [157,158,159]. As with most components of the immune system, inflammatory responses must be tightly regulated.

## 4. The Benefit of Understanding Bony Fish Immunology

Throughout history, human beings have relied on fish populations for sustenance. With regard to the environment, fish play a vital role in natural food webs by supporting the growth and survival of numerous species [160,161]. From an economic perspective, fish represent a valuable commercial product providing many career options while also ensuring that people have access to healthy protein sources worldwide. Aquaculture production also has the added benefit of ensuring that wild populations will not be dangerously overfished in order to meet the rising demand for this food source. As the intensive culture of fish is still in its infancy when compared to terrestrial agriculture, many developments still need to be made. One way to help enhance the productivity of aquaculture efforts is to obtain a deeper understanding of teleostean immunity. Advances here will lead to improved vaccine design and therapeutic options, thereby augmenting yields and strengthening fish health in these facilities. 

Although there are many similarities between the immune system of fish and mammals, there are also notable differences. Despite the highly studied mammalian model providing a solid baseline, comparative immunologists must experimentally confirm whether mammalian immune responses and tissues are analogous in fish. As an example, the bone marrow of mammals is the site of haematopoiesis and where B cells develop (reviewed in [162]). However, fish do not have bone marrow, so instead this essential process occurs in the anterior portion of their kidney (reviewed in [163]) but research is still being conducted to fully understand this process in teleosts. When it comes to specific immune receptors, fish have many similarities to those found in mammals (reviewed in [164]). Nevertheless, teleostean equivalents quite often vary in number and ligand specificity. This can be seen when comparing TLRs between these two taxa. One mammalian example, human, is known to have 10 different TLRs, while at least 20 have been found in more than a dozen fish species (reviewed in [48,49,165]). Some of these human TLRs can be found in fish, while others have been lost in many bony fish species, such as TLR6 and TLR10 (reviewed in [165]). Despite these interesting differences between fish and mammalian TLRs, direct evidence of ligand specificity has only been shown in seven of the twenty TLRs found in fish [52,55,166,167]. Given the large differences in environment, combined with the fact that fish have undergone at least one, if not two, whole genome duplication (WGD) events not experienced by mammalian species (reviewed in [15]), it is not surprising that there are significant differences both functionally and genetically between fish and mammals. This emphasizes the importance of validating immune paradigms in teleosts before basing therapies on concepts that have only been confirmed in mammalian models. 

Teleosts represent approximately half of all surviving vertebrate species, making them the largest and most diverse group of this subphylum [168]. Just as there are differences between species within the taxa Mammalia (reviewed in [169,170]), there are also distinct differences between species of fish, and indeed these differences are bigger given that fish emerged and diversified close to 400 million years ago (reviewed in [171]). As an example, the majority of studied fish have genes for both MH class I and MH class II molecules but the Atlantic cod (*Gadus morhua*) has lost the genes for MH class II and its accessory molecules [172]. Furthermore, the cod-like fish, broadnosed pipefish (*Syngnathus typhle*), has lost MH class II function while still maintaining some of the genetic information [173]. When the whole genome of cod was compared to other sequenced fish species, it was observed that cod has large gene expansions and several gene losses in the TLR repertoire [174]. It is possible that the loss of such a large component of adaptive immunity resulted in a greater dependence on the innate immune system in this particular species. Understanding interspecies differences in immunity such as these will help increase the efficacy of future therapies and vaccine initiatives. 

## 5. Current Methods of Bacterial Disease Prevention in Aquaculture

### 5.1. Heritable Differences in Selectively Bred Fish

There are a number of ways in which the breeding of fish differs from other livestock, and this is due to the often-high fecundity of aquatic species. This allows for a strong selection intensity and results in large families, which can facilitate the extensive collection of the phenotypic records of close relatives for selection candidates within breeding programs (reviewed in [175]). Since it relates directly to the economic potential of an aquaculture facility, growth is often the initial focus of selective breeding endeavors. Heritability of growth has been observed in several fish species including tilapia (*Oreochromis niloticus*), rainbow trout (*Oncorhynchus mykiss*), Atlantic salmon (*Salmo salar*), Asian seabass (*Lates calcarifer*), as well as many others [176,177,178,179]. Though now well established, the first breeding programs for aquatic species used mass selection and were generally unsuccessful [180,181]. It is now understood that this was likely due to the accumulated effects of inbreeding as they are known to influence several economic traits including growth [178,182,183,184]. Fortunately, with the development of creative breeding programs, a reduction in inbreeding can be consistently predicted for some aquatic species [185]. However, because the effects of heterosis tend to vary between species and individual stocks, confirmation of genetic improvements should be analysed on a case-by-case basis. 

Despite being extremely valuable, growth is certainly not the only factor that is selected for in aquaculture. Given the large losses due to infectious disease [5], many breeding programs have been adopted to maintain improved growth while also selecting for resistance to problematic pathogens (reviewed in [186]). In some cases, heritability of disease resistance is observed without negatively influencing animal growth [187], such as has been observed in rainbow trout with resistance towards *F. psychrophilum* [188] or *F. columnare* [189]. However, it has also been shown that selecting for resistance to a single pathogen can sometimes result in increased susceptibility to another or in decreased growth [190,191,192,193,194]. In these studies, the gold standard for determining disease resistance is survival comparisons during live infection challenges (reviewed in [195]). Unfortunately, the subsequent results of these challenges may not be representative as experimental infection models are often not comparable to what would be observed in a natural infection. For some aquatic pathogens, experimental models of infection that appropriately mimic live infection have still not been established. This has led to several alternative approaches for mimicking the outbreaks that are observed in aquaculture facilities, such as co-habitation [196,197], waterborne [198,199,200] and stress induction challenges [201,202]. The major issue with these approaches is that they are often not repeatable if they are successful in producing disease symptoms. Intraperitoneal injection is often used and will ensure a much more consistent infection status for disease challenge trials. However, this method is not representative of a natural outbreak as the integument and mucous barriers are bypassed. The lack of appropriate challenge models has been a confounding factor for determining whether disease resistance observed in a lab setting will translate to disease resistance in an intensive culture situation. Understandably, this has also made it difficult to determine the true efficacy of vaccines and other therapeutics prior to their use in aquaculture facilities. 

In an attempt to make salmonid stocks that are more robust and/or resilient in the face of bacterial disease, several breeding strategies have been pursued. A typical first attempt is selective breeding or artificial selection, wherein plants/animals presenting desirable traits, such as resistance to infectious disease, are used in breeding regimes so that the next generation will present these traits at a higher frequency [12]. One example of this can be seen when trying to select for resistance in rainbow trout against *F. psychrophilum*, the causative agent of bacterial coldwater disease (BCWD). Because this condition primarily affects animals at a young age, different families initially present markedly variable survival to BCWD. However, as the fish grow larger in size this variability in survival appears to be lost when receiving i.p. injections with *F. psychrophilum* [203]. This phenomenon of age-related disease susceptibility has been reported with other aquatic diseases including *F. branchiophilum* [204] and infectious pancreatic necrosis virus (IPNV, [205]). However, several long-term breeding studies have shown that resistance to BCWD is heritable and also that breeding over multiple generations can achieve consistently high resistance [206,207,208]. Though promising, this is a large and costly undertaking that would be difficult to perform for every bacterial disease of interest in aquaculture and also only offers potential protection against a single pathogen. The functional immune components responsible for the observed resistance to BCWD have not yet been identified, though there is some evidence to support the hypotheses that MH class IB [209], spleen size [210], IgT^+^ B cells [211] and the microbiome of mucosal tissues [212] could be implicated. However, because correlation is not always causation, some of these examples have already been debunked [213], emphasizing the importance of trial repetition and design in future experiments. 

### 5.2. Vaccinations and Their Efficacy

Although there are several different types of vaccines produced for aquaculture (reviewed in [214]), the majority that are used for bacterial pathogens are killed whole-cell preparations. These do provide some protection but when compared to the successes of terrestrial vaccine formulations, are limited at best. This lower efficacy is likely due to fundamental differences between the teleostean and mammalian adaptive immune response and how it translates into immunological memory, as described above in Section 3.2.3. Aside from differences in the functional immune response, which is still not fully understood, the environment of teleosts likely plays a significant role. Temperature has in particular been shown to drastically influence the binding kinetics of monoclonal antibodies produced from terrestrial organisms in vitro [215,216,217]. Indeed, temperature has been explored in vivo in various teleost species and has been shown to influence antibody development to a wide array of antigens, with lower temperatures generally associated with a decrease in antibody production [218,219]. It is well known that higher temperatures provide more energy, often leading to an accelerated interaction between ligands and targets. Because the majority of salmonid species are cold water fish, and therefore also have lower body temperatures, it would stand to reason that this would repress antibody production when compared to mammalian counterparts. However, this does not explain the comparably slow antibody development also observed in warmwater teleosts [219,220,221,222,223]. Though still slower than what is observed in the majority of terrestrial organisms, higher temperatures are generally associated with faster development and/or a higher antibody titer in salmonids [224,225,226,227]. Curiously, the opposite trend has been reported in some warmwater teleosts, as was seen in catfish (*Ictalurus punctatus*) following vaccination to *Edwardsiella ictaluri* bacterin. When held at a higher temperature (25 °C) for 60 days, the catfish presented lower antibody titers when compared to their counterparts held for 30 days at 25 °C, followed by 30 days at 12 °C [222]. If antibody production is directly related to physiological temperature, the opposite trend would have been anticipated. Thus, although temperature likely does play a role, it is not the sole explanation for the delayed immune response associated with teleostean antibody development and immunological memory. 

Rather than the rapid isotype switching following an initial exposure to a foreign entity, fish rely heavily on both IgM and IgT, antibody responses (reviewed in [120]). This reveals the importance of stimulating mucosal immunity for future vaccine design regimes, something that was not a central focus historically (reviewed in [228]). For certain bacterial pathogens, an immersion vaccination of fish has been found to be effective in inducing a mucosal response, as has been observed for *V. anguillarum* and *F. psychrophilum* [229,230]. However, in many of these cases boosters are required and application routines including those have not been developed for cultured fish (reviewed in [231]). In mammals, high antibody levels can be observed as early as two weeks following antigen exposure in what is known as the primary antibody response [41]. Yet based on recent data, even after four weeks and/or four months following live infection, the reported variability in teleost antibody production is high, with many individuals presenting low to negligible antibody production [203,232] and with a large variation in response even within the same family of fish [233]. This low level of antibody production is consistent with what has been observed previously in other teleostean models where antibody titers can require 12 weeks or longer to reach peak levels in rainbow trout [234,235]. Given that delayed antibody production is a consistent finding in teleosts, perhaps alternative approaches must be made to successfully stimulate protective responses towards bacterial pathogens. Importantly, this would require a deeper understanding of adaptive immunity in relevant teleostean species.

### 5.3. Understanding Bacterial Pathogens and the Resulting Disease State

Surprisingly, despite the negative impact that bacterial pathogens have on aquaculture facilities, there is limited research devoted to understanding the pathogenesis of these organisms. Elucidating the pathologic cycle has been instrumental in developing effective vaccines for several terrestrial pathogens. The vaccines for both tetanus (*Clostridium tetani*) and diphtheria (*Corynebacterium diphtheriae*) are toxoids as it is the toxin produced by the bacterium that causes fatalities, not the organism itself [236]. The influence of extracellular products on disease state has been observed with aquatic pathogens in vitro as has been reported with numerous bacteria including: *F. psychrophilum* [237], *Pasteurella piscicida* [238], *A. salmonicida* [239,240,241], and *Moritella viscosa* [242,243]. Furthermore, these observations have been translated to in vivo studies as was observed when infecting Atlantic salmon with *M. viscosa* [244] or when exposing rainbow trout to the extracellular products of *A. salmonicida* [245]. Interestingly, the conditioned media of *F. psychrophilum* alone has been shown to stimulate cytokine proinflammatory transcript levels in rainbow trout immune cells and to significantly inhibit their early phagocytic activity [237]. Indeed, the extracellular products of some aquatic bacteria have historically been a focus regarding their pathogenesis. Ostland and colleagues previously demonstrated that when a crude extracellular preparation of *F. psychrophilum* was injected directly into the muscle of rainbow trout, muscle necrosis was observed [246]. This led to further studies attempting to determine virulence factors of *F. psychrophilum*, including the identification of extracellular proteases and mutation analyses using the identified secreted proteases [247,248,249,250]. Unfortunately, identification of individual virulence factors has had limited success, but loss of virulence has been observed in some cases [247,251]. Removal of predicted virulence factors can result in a decrease in virulence for aquatic bacterial pathogens in vitro [252,253,254], but this is not always the case [255,256]. The majority of these experiments focus solely on the resulting virulence of the pathogens of interest. Some analyses have observed the immune function of the host, but these represent a small number in the field due to the high associated financial costs and time commitments. Therefore, studies that explore the interaction of bacterial pathogenesis with the host immune system are required to develop improved and efficient treatment options.

Like many terrestrial pathogens, it appears that some aquatic bacterial pathogens have both intracellular and extracellular components to their pathologic cycles [257,258,259,260,261,262,263,264]. This may further impede the design of some antibacterial therapies as vaccines for well-studied intracellular terrestrial pathogens, such as *Mycobacterium tuberculosis*, have shown variable efficacy (reviewed in [265,266]). In these situations, a typical humoral response will not successfully eliminate the pathogen of interest, so alternative methods of immune stimulation must be developed (reviewed in [267,268]). This mirrors the difficulties and variable success observed when attempting to develop a fish vaccine for *R. salmoninarum*, a known aquatic intracellular bacterial pathogen [269,270]. Similarly, it is believed that *F. psychrophilum* has both intracellular and extracellular aspects of its pathologic cycle, making vaccine development difficult [263,271]. Despite all that is unknown regarding *F. psychrophilum*, some promising vaccine candidates have been developed recently for BCWD [272,273,274], but these studies must be repeated and tested during natural outbreak/exposure conditions. For many bacterial pathogens, developing an effective vaccine or improving current formulations has required an understanding of the bacterial pathogenesis. This is precisely the information that is lacking with regard to many salmonid pathogens afflicting aquaculture and is also why studies should aim to understand the pathologic cycle of these pervasive organisms.

### 5.4. Adjuvants/Immunostimulants for Aquaculture

As seen in successful mammalian vaccine preparations, the presence of the antigen itself is important driving the specificity of immunological memory, but appropriate adjuvants are key to ensure that the resulting immune response is protective. If a vaccine contained purified protein antigens alone, this would result in only a slight antibody response with little to no T cell activation. Thus, multiple immunizations would likely be required to stimulate a sufficient antibody memory response (reviewed in [275]). For terrestrial animals, there are a variety of different and effective adjuvants used for vaccination programs (reviewed in [276]). Presently for fish, commercial vaccines consist mainly of purified antigens for the pathogen of interest as well as an emulsifying agent (reviewed in [277]). However, there has been significant research regarding the use of PAMPs, cytokines and other immunostimulants to enhance vaccine efficacy. For many of these studies, the protective response has been improved during experimental challenge with the pathogen of interest. The addition of flagellin along with Hsp60 and Hsp70 chaperonins to a subunit vaccine for *P. salmonis* resulted in a high protective response in Atlantic salmon [278]. One study used aluminum sulphate (alum), a common adjuvant of mammalian vaccines, in conjunction with an *Escherichia coli* mutant, to vaccinate for *E. ictaluri* in catfish. Following the disease challenge, the alum adjuvant resulted in 92% survival when compared to the 54% survival observed with the no adjuvant control [279]. Adjuvants have been an essential component in the successes of mammalian vaccines. Their addition to vaccine candidates for fish have had promising results, but further study is required to determine whether these results are reliable and/or reproducible.

To help promote an appropriate type of immune response, recombinant cytokines can also be added to vaccine preparations to act as adjuvants. With vaccines for teleost species, this is a relatively new undertaking that has shown some promising results. When recombinant IL-8 was added to a vaccine for *Streptococcus iniae*, catfish showed a 20% greater survival four weeks following the challenge with the pathogen [280]. However, this did not appear to provide long-lasting immune protection as survival did not differ significantly from the control at week eight [280]. Cytokines as adjuvants can also be used to strategically direct an immune response in aquatic species. The addition of IL-12 to a *Nocardia seriolae* vaccine was able to suppress humoral immunity and stimulate a much more protective cell-mediated response in juvenile amberjack (*Seriola dumerili*), resulting in an increase in survival by an impressive 88% when challenged with the bacterial pathogen [281]. Although not in combination with an actual vaccine preparation, rainbow trout receiving intraperitoneal injections of recombinant IL-1β presented a significantly increased survival to *A. salmonicida* as well as total leukocyte number in peritoneal exudates [282]. Aside from the physical addition of adjuvants to vaccine preparations, the host machinery can be manipulated to produce recombinant cytokines. This is accomplished through the injection of expression plasmids that contain DNA sequences for the desired proteins. Using these DNA vaccines, host-made cytokines such as IL-8 and IL-1β have shown promise as adjuvants in flounder, sea bass and rainbow trout [283,284,285,286], but have yet to be tested in aquaculture outbreak situations. Despite these promising indications, the use of recombinant salmonid cytokines requires further exploration. Unfortunately, there remains a lack of assays specifically targeting teleost immune proteins [287]. Thus, our understanding of these important immune modulators is primarily limited to the genomic level. 

The production of AMPs by fish species has been extensively studied as an alternative treatment for antibiotics (reviewed in [288,289]). Aside from their direct impact on pathogens, AMPs have been shown to significantly influence the immune system of fish, making them prime candidates as indirect therapeutic agents and/or adjuvants. In fact, co-administration of AMPs with antigens from pathogens has been shown to boost immunogenicity in tilapia [290]. In salmonids, there have been few studies involving the co-administration of AMPs with vaccine candidates, but exposure to AMPs alone has been consistently shown to stimulate proinflammatory responses in vitro and in vivo [291,292,293]. There have also been examples of AMPs protecting salmonids in vivo when challenged with live bacterial pathogens [294]. Similarly, when fish are challenged with bacterial pathogens or relevant antigens, a significant induction of antimicrobial peptides in response to these stimuli has been reliably observed [295,296,297,298], indicating the defensive nature of these peptides. Teleostean AMPs have also shown synergistic effects when used in conjunction with other AMPs [299] or when used with therapeutic drugs [300] in laboratory settings. An example of this has been noted with pituitary adenylate-cyclase activating polypeptide (PACAP). When PACAP was administered in the presence of a bacterial pathogen, the AMP significantly enhanced the stimulation of proinflammatory transcripts [301]. When used alone, PACAP was able to significantly stimulate proinflammatory transcripts of rainbow trout immune cells, indicating that this peptide has an immunostimulatory activity similar to other studied mammalian AMPs [302,303,304]. Interestingly, these stimulatory effects are contrary to what has been observed with PACAP in mammalian studies wherein anti-inflammatory responses have been primarily observed [305,306,307]. This emphasizes the importance of confirming the activity of these molecules in each model organism before assigning a canonical function. Additionally, as a member of the secretin/glucagon/growth hormone-releasing hormone/vasoactive intestinal peptide superfamily [308], PACAP has also been linked to improved growth in some fish species [309,310,311], thereby adding another potential benefit for farmers if used as an adjuvant and/or a therapeutic agent. Given the need for appropriate adjuvants in fish vaccines, the use of AMPs may provide an effective, environmentally friendly alternative that is naturally produced by the host species.

This review highlights the negative impacts of bacteria in aquaculture settings, but it is important to acknowledge that certain bacteria can actually be beneficial for cultured aquatic species. This can be seen in studies investigating the use of probiotics as potential vaccine adjuvants and/or immunostimulants in aquaculture (reviewed in [312]). Probiotics are non-pathogenic microorganisms that, when ingested, exert a positive influence on the health or physiology of the host [313]. In rainbow trout, there have been several studies revealing that probiotic bacteria can prevent the colonization and growth of bacterial pathogens and/or enhance the survival of the host to *F. psychrophilum*, *V. anguillarum*, *Y. ruckeri*, and *A. salmonicida* infections [314,315,316,317,318,319,320,321,322,323,324]. Similar results have been presented in other salmonid species including brown trout (*Salmo trutta*) [325] and Atlantic salmon [326,327]. Additionally, dietary supplementation with probiotics has been shown to improve the efficacy of vaccine candidates in rainbow trout [328,329,330], outlining their potential as adjuvants. Though the mechanisms responsible for these promising biological effects are not fully understood, there is significant evidence indicating that some probiotics can stimulate leukocyte phagocytic activity [316,331], IgT expression [332,333], and inflammatory cytokine expression [332,334,335]. Moreover, because probiotics are generally administered in feed, the use of these treatments can often provide protective effects on the gut epithelium and towards gut immunity [333,336,337,338]. This was observed in Atlantic salmon when infection with *V. anguillarum* and *A. salmonicida* resulted in extensive damage to the intestinal mucosal epithelium, yet the same exposure to a probiotic strain of bacteria resulted in histological presentations that were indistinguishable from control fish [337]. Furthermore, when these fish were co-inoculated with pathogenic and probiotic bacterial strains, the damage to the intestinal mucosal epithelium was reduced or completely abolished [337]. Interestingly, alternative administration routes of probiotics have also shown favorable results, such as when Atlantic salmon received a probiotic bath which resulted in enhanced growth and decreased mortality/morbidity during a natural outbreak of *M. viscosa* [339]. The use of probiotics in aquaculture as a potential adjuvant/immunomodulator shows promise but further research is necessary to fully understand the mechanisms of action, optimal administration routes and potential side-effects from long-term use.

### 5.5. Alternative Study Models—The Value of Fish Cell Lines for Immune Analyses

Considering the diversity of teleostean species and their utility all over the world, relatively few cell lines are available to represent the many members of this infraclass, especially when compared to mammals. The first permanent fish cell line, RTG-2, was established in 1962 from rainbow trout gonadal tissue [340]. Since then, more fish cell lines have been created with more than 300 being developed from approximately 110 species of teleosts (reviewed in [341,342]). This only accounts for 0.4% of the known 26,000 teleostean species. Many of these cell lines are derived from industrially relevant species but as aquaculture practises change to meet market demands/preferences, the establishment of appropriate eukaryotic cell lines is necessary. With the rising interest in global salmonid aquaculture, the development of cell lines from relevant tissues and species will ensure that researchers have the necessary tools to explore various aspects of cellular function, physiology and immunity in these valuable species. 

Historically, the primary purpose of fish cell lines has been to isolate, propagate and study aquatic viruses. Though less common, these cultures can also provide a controlled, cost-effective environment to explore both the pathogenesis and host cellular immune response associated with bacterial pathogens. This has been observed with several aquatic bacterial pathogens including *Y. ruckeri* [343], *Francisella noatunensis* [344], *F. psychrophilum* [237], *V. anguillarum* [345] and *Photobacterium damselae* [346]. These and other similar studies were able to significantly improve the understanding of the associated pathogens and helped to improve future in vivo trials. An analysis of *P. salmonis* using the rainbow trout spleen macrophage/monocyte-like cell line, RTS11, revealed important information regarding the immunomodulatory effects of this bacterium. This provided a possible explanation for why previous vaccine attempts were not stimulating protective responses in salmonids. Infection with *P. salmonis* results in an upregulation of the anti-inflammatory cytokine, IL-10, as well as a reduced amount of the antimicrobial peptide, hepicidin [262]. Additionally, vesicles containing hepicidin were unable to merge with the bacterium, rendering the bactericidal peptide useless [262]. Such detailed analyses of cellular interactions with bacterial pathogens could potentially be overlooked without the use of relevant fish cell lines. 

Many species of teleost fish, particularly salmonids, have complex life cycles involving an early freshwater phase followed by a transition to a saltwater environment. This transition entails not only dramatic physiological changes that the animals have to undergo, but also exposure to pathogens present in both salt- and freshwater habitats. As such, it is unsurprising that we observe important differences in immune performance between salmonids from different life-stages [347]. This emphasizes the importance of developing cell lines, not only from different species, but also from different life stages to accurately represent relative immune responses. Several in vitro studies have been conducted with the saltwater pathogen, *V. anguillarum*, in salmonid cell cultures [348,349,350,351,352], revealing important properties regarding the virulence of this organism. As a common bacterial pathogen on the Pacific coast of North America, there has been previous in vitro work with this organism using the embryonic Chinook salmon (*Oncorhynchus tshawytscha*) cell line, CHSE-214 [345,350,353], but these studies primarily looked at properties of the bacterium itself with little study of cellular host immunity. Until recently, CHSE-214 was the only cell line derived from Chinook salmon, a native salmonid of the Pacific coast. However, with the recent development of CHSS [354] and CHST [232], studies can now be conducted using Chinook cell lines from adult fish. Furthermore, it was shown that the adult CHSS cell line contained immune receptors that were found to be absent on the embryonic CHSE-214 cells [354]. As previously shown in multiple studies, the developmental age of the animal can alter the immune defenses observed, even in the resulting cell lines produced [347,354,355]. Because the most significant economic losses in aquaculture are experienced with market-sized fish, cell lines from adult tissue donors would be very applicable for this industry. However, great numbers of losses can also occur during juvenile stages, so cell lines representing all life stages could provide interesting insights for aquaculture production. 

Eukaryotic cell lines are very useful for biological experimentation, but these tools should not be perceived as a replacement for whole animal research. In situations where in vivo studies are not possible or there are technological limitations, cell lines can provide preliminary results that are hypothesis generating. However, it is important to recognize that each cell line represents one cell type from a single individual, and thus these model systems cannot be expected to perfectly represent whole animals or processes that require the interactions of multiple cell types like immune responses, let alone entire populations. This is supported by numerous studies wherein mammalian cell lines have revealed that passage number and culture time can influence gene expression, enzyme activity, morphology and cell reactivity/responsiveness [356,357,358,359,360,361,362]. Although there has been little study cataloging this disconnect in teleost cell lines, it undoubtedly entails similar limitations. 

## 6. Concluding Remarks

The vertebrate immune system is a complicated network, consisting of molecules with complimentary and antagonistic actions, interacting cells, and cell-forming tissues that all work together in an effort to protect the body from infectious agents and other foreign substances. The complexity of the immune system did not arise spontaneously but rather is the result of over 500 million years of evolution. Over this time, the immune system has continuously evolved between taxa, maintaining components that are valuable while removing others that are of little use. Aquatic and terrestrial organisms occupy a wide variety of different habitats and are therefore subject to different evolutionary pressures. As such, the composition of the immune system between species can vary widely, reflecting these environmental differences. Thus, it is no surprise that we have been able to use comparative models to (a) enhance our understanding of mammalian immunity, (b) improve animal husbandry in a species-specific way and (c) discover useful immunological tools for molecular biology applications. This review has attempted to catalogue some of the more recent findings within teleostean antibacterial immunity, and due to enhanced interest in the culture of aquatic species, this field is rapidly expanding.

With the demand for salmonid culture continually increasing, treatment options to combat infectious disease outbreaks are invaluable. Specifically, for contagions of bacteria, the current approach is the use of antibiotics, despite the tight regulations limiting their use to prevent the development of antibiotic resistance. As a result, antibiotics are not a sustainable method of choice despite their efficacy in containing bacterial disease outbreaks in real time. Gaining a deeper knowledge of salmonid immunity is essential for streamlining some of the promising alternative methods for promoting disease resistance, such as through breeding strategies to select/promote heritable markers. In situations where this is not possible, improving prophylactic treatments or optimizing alternative treatment options/potential adjuvants, such as AMPs or probiotic bacterial supplementation, could provide environmentally friendly options for bacterial disease containment. Additionally, by learning more about the pathologic cycle of problematic bacterial pathogens and also what constitutes an effective immune response in the host, researchers would be able to tailor treatment regimes, enabling the development of new and improved vaccines. This can be accomplished not only through large and costly in vivo trials but also through improvements and developments to applicable cell lines, so that the host cellular immune response can be further elucidated. All of this has the potential to significantly reduce a large portion of the $6 billion dollars of disease losses in the global aquaculture industry. Salmonid aquaculture is an important industry, and as such, research meant to provide alternative treatment options for bacterial disease outbreaks is invaluable for helping to ensure the sustainability of this expanding industry.

## Figures and Tables

**Table 1:** Common bacterial pathogens in salmonid aquaculture. A list of bacterial pathogens that affect the aquaculture production of salmonids, their Gram reaction and the diseases/conditions that they are associated with. Several of these conditions will be discussed throughout this review in order to describe various methods used in aquaculture to prevent and/or treat. The “−” symbol denotes a negative Gram reaction while the “+” symbol denotes a positive Gram reaction.

Bacterial Species	Gram Stain Reaction	Disease
*Yersinia ruckeri*	−	Enteric Redmouth Disease
*Flavobacterium columnare*	−	Columnaris Disease
*Flavobacterium psychrophilum*	−	Bacterial Coldwater Disease (BCWD)
*Flavobacterium branchiophilum*	−	Bacterial Gill Disease (BGD)
*Moritella viscosa*	−	Winter Ulcer
*Edwardsiella tarda*	−	Edwardsiellosis
Piscirickettsia salmonis	−	Piscirickettsiosis
*Aeromonas salmonicida*	−	Furunculosis
*Aeromonas hydrophila*	−	Motile Aeromonas Septicemia
*Tenacibaculum maritimum*	−	Mouthrot
*Vibrio salmonicida*	−	Hitra Disease, Coldwater Vibriosis
*Vibrio veronii*	−	Epizootic Ulcerative Syndrome
*Vibrio anguillarum*	−	Vibriosis
*Renibacterium salmoninarum*	+	Bacterial Kidney Disease (BKD)
*Mycobacterium marinum*	+	Mycobacteriosis
*Streptococcus phocae*	+	Streptococcosis
